# Dental pulp-derived stem cells can counterbalance peripheral nerve injury-induced oxidative stress and supraspinal neuro-inflammation in rat brain

**DOI:** 10.1038/s41598-018-34151-x

**Published:** 2018-10-25

**Authors:** Imran Ullah, Yong-ho Choe, Mehtab Khan, Dinesh Bharti, Sharath Belame Shivakumar, Hyeon-Jeong Lee, Young-Bum Son, Yurianna Shin, Sung-Lim Lee, Bong-Wook Park, Sun-A Ock, Gyu-Jin Rho

**Affiliations:** 1 Department of Theriogenology and Biotechnology, College of Veterinary Medicine, Gyeongsang National University, Jinju, Republic of Korea; 2 Research Institute of Life Science, Gyeongsang National University, Jinju, Republic of Korea; 3 Animal Biotechnology Division, National Institute of Animal Science, Rural Development Administration, 1500 Kongjwipatjwi-ro, Isero-myeon, Wanju-gun, Jeollabuk-do, 565-851 Republic of Korea; 4 Department of Oral and Maxillofacial Surgery, Changwon Gyeongsang National University Hospital, Changwon, Republic of Korea; 5 Department of Dentistry, Gyeongsang National University School of Medicine, Institute of Health Science, Jinju, Republic of Korea; 6 Department of Biology and Applied Life Science (BK 21plus), College of Natural Sciences, Gyeongsang National University, Jinju, 660-701 Republic of Korea

## Abstract

Previously, we reported the successful regeneration of injured peripheral nerves using human dental pulp stem cells (DPSCs) or differentiated neuronal cells from DPSCs (DF-DPSCs) in a rat model. Here, we attempted to evaluate oxidative stress and supraspinal neuro-inflammation in rat brain after sciatic nerve injury (SNI). We divided our experimental animals into three SNI groups based on time. The expression of a microglial (Iba1) marker and reactive oxygen species (ROS) was lower in DPSCs and higher in DF-DPSCs. In contrast, the expression of an astroglial (GFAP) marker was higher in DPSCs and lower in DF-DPSCs at 2 weeks. However, the expression of ROS, Iba1 and GFAP gradually decreased at 8 and 12 weeks in the SNI DPSCs and DF-DPSCs groups compared to the SNI control. Furthermore, anti-inflammatory cytokine (IL-4 and TGF-*β*) expression was lower at 2 weeks, while it gradually increased at 8 and 12 weeks after surgery in the SNI DPSCs and DF-DPSCs groups. Similarly, SNI DPSCs had a high expression of pAMPK, SIRT1 and NFkB at the onset of SNI. However, 12 weeks after surgery, pAMPK and SIRT1 expression levels were higher and NFkB was down-regulated in both DPSCs and DF-DPSCs compared to the control group. Finally, we concluded that DPSCs responded early and more efficiently than DF-DPSCs to counterbalance peripheral nerve injury (PNI)-induced oxidative stress and supraspinal neuro-inflammation in rat brain.

## Introduction

Mesenchymal stem cells (MSCs) are considered one of the most important cell types for regenerative medicines. MSCs can migrate to the site of injury, where they initiate immune and inflammatory responses through several paracrine mechanisms^1^. To date, MSCs have been successfully isolated from numerous tissue sources, including adipose tissue, bone marrow, umbilical cord, and dental tissues^2^, and have distinct differentiation characteristics. Previously, we reported the isolation of MSCs from different dental tissues^3^ that successfully regenerated injured peripheral nerve in a rat model^4^. Peripheral nerve injury (PNI) causes neuropathic pain that can be acute or chronic depending on the severity of the injury^5^. Clinically, neuropathic pain is characterized by sensory and affective disturbances. Sensory disturbances comprise hyperalgesia, spontaneous pain, dysesthesia and paraesthesia, while affective disturbances comprise impaired cognition, stress, depressive-like behavior, sleep disturbances and disturbed social interactions^6–10^. Sensory and affective disturbances are an outcome of interactions between neurons and immune cells (immune cell-derived inflammatory mediators), particularly chemokines and cytokines as well as histamine, ATP and prostaglandins^11–13^. Hence, neuropathic pain is called a neuro-immune disorder^14^. PNI induces the release of certain inflammatory mediators especially at the site of injury and at the dorsal root ganglia (DRG) as well as at the spinal cord recipient segments^14^. Moreover, peripheral neuropathy also induces the release of inflammatory mediators within the brain, but surprisingly little attention has been paid to this supraspinal neuro-inflammation^14^. Neuropathic pain causes anxiety and depressive-like behavior in patients as well as neuropsychiatric disorders, which are associated with the release of pro-inflammatory cytokines and chemokines^15–17^. Patients suffering from affective disturbances, i.e., depression and stress, have a strong inflammatory component with higher levels of blood pro-inflammatory cytokines, such as interleukin-6 (IL-6) and tumor necrosis factor (TNF)^18,19^. Furthermore, Uceyler *et al*. (2007) reported that patients with neuropathy followed by depression have higher TNF levels than patients without depression^20^. These observations suggested that this mixed composition of pain and depression share a common inflammatory mechanism^21^. The release of inflammatory mediators that cause supraspinal inflammation may occur in response to the local environment in the brain but also due to the release of peripheral cytokines initiated by a distant inflammatory event^22^. Along with inflammation, affective disturbances also lead to stress conditions resulting in a higher expression of reactive oxygen species (ROS) and energy depletion. Adenosine monophosphate kinase (AMPK, main energy regulator) and oxidative stress have long been associated with neurodegenerative diseases^23^. The main target of ROS is mitochondria, which leads to disruption of mitochondrial respiration and ultimately apoptotic cell death and degeneration^24^. Similarly, chronic inflammation also activates microglia (Iba1) and the release of pro-inflammatory cytokines accompanied by ROS, resulting in neuro-degeneration and synaptic transmission alterations^25^. Activation of AMPK/SIRT1 enhances energy production, resulting in the inhibition of nuclear factor kappa activated B cells (NFκB) and subsequently pro-inflammatory cytokine production^25^.

In the present study, we evaluated the relieving effect of dental pulp stem cells (DPSCs) and neuronal differentiated DPSCs (DF-DPSCs) on peripheral nerve injury-induced neuro-inflammation in the brain of a sciatic nerve injury (SNI) rat model.

## Results

### Microglial and astroglial activation

**Figure 1:**
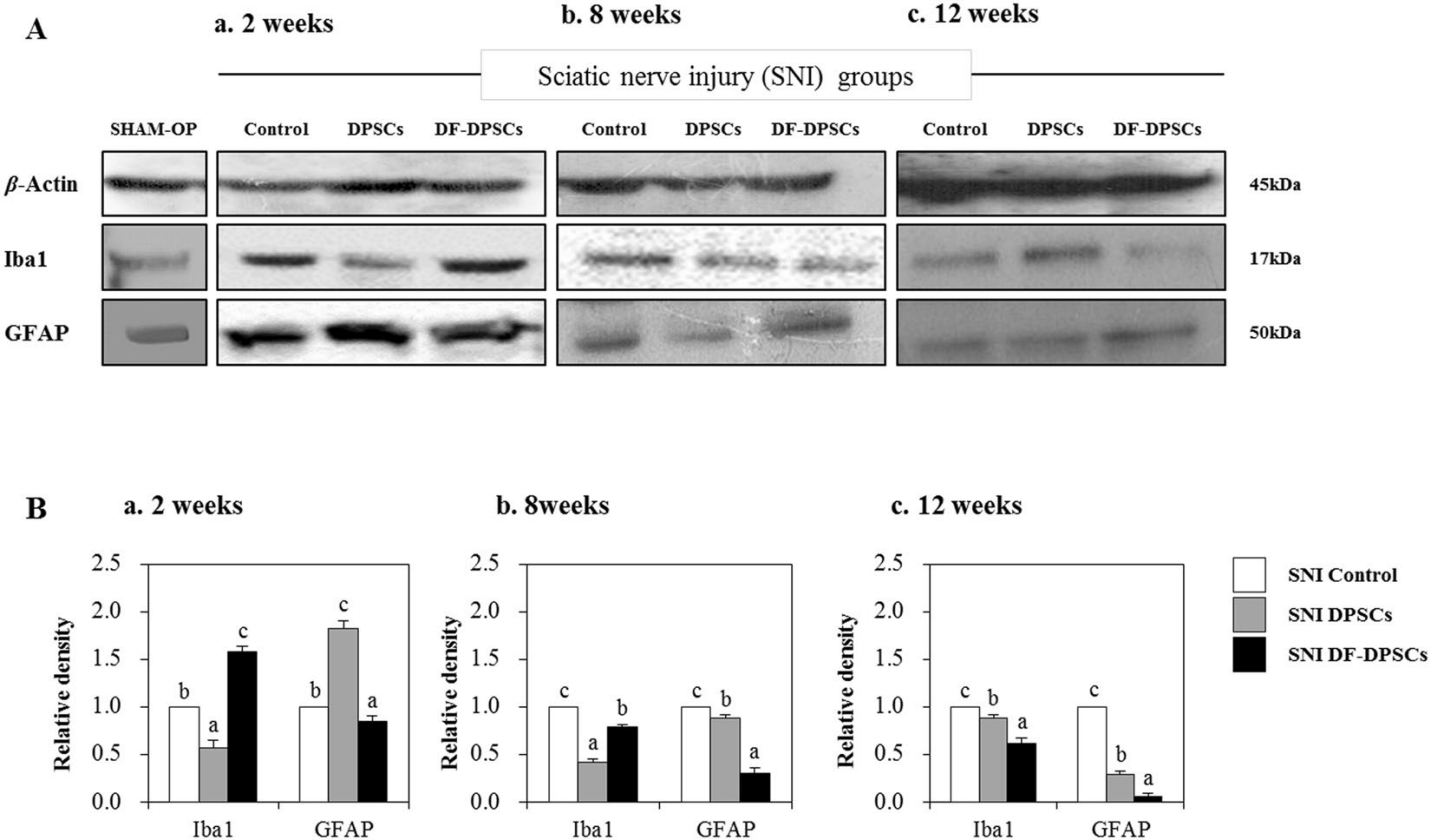
Western blot analysis of microglia/astroglial markers in rat brain 2, 8 and 12 weeks after surgery. Western blot analysis of Iba1 and GFAP (**A**a) 2 weeks, (**A**b) 8 weeks, and (**A**c) 12 weeks after surgery. Integrated density histograms of Iba1 and GFAP after 2, 8 and 12 weeks (**B**a–c), respectively. *β*-Actin was used as an internal control. The characters a, b, and c indicate significant differences (*p* < 0.05) among the SNI groups. SHAM OP represents the normal control.

Microglial (Iba1) and astroglial (GFAP) expression was evaluated in SNI groups by Western blot 2, 8 and 12 weeks after surgery. After 2 weeks, we found low expression of Iba1 and high expression of GFAP in SNI DPSCs compared to other groups, while SNI DF-DPSCs had inverse results (Fig. 1Aa,Ba). After 8 weeks, SNI DF-DPSCs had low expression of Iba1 compared to controls but higher expression than DPSCs while the expression of GFAP was further decreased in DPSCs (Fig. 1Ab,Bb). After 12 weeks, both DPSC groups had significantly low expression of Iba1 and GFAP compared to controls, though DF-DPSCs had the lowest expression among the three groups (Fig. 1Ac,Bc). The expression of Iba1 and GFAP was also measured in a SHAM OP group, which was comparable with those of the SNI DPSCs and DF-DPSCs groups 12 weeks after surgery (Fig. 1Aa). However, the expression was lower than all SNI groups at 2 and 8 weeks after surgery. Figure Ba-c showed representative relative band density histograms.

### ROS and TGF- *β* activity

**Figure 2:**
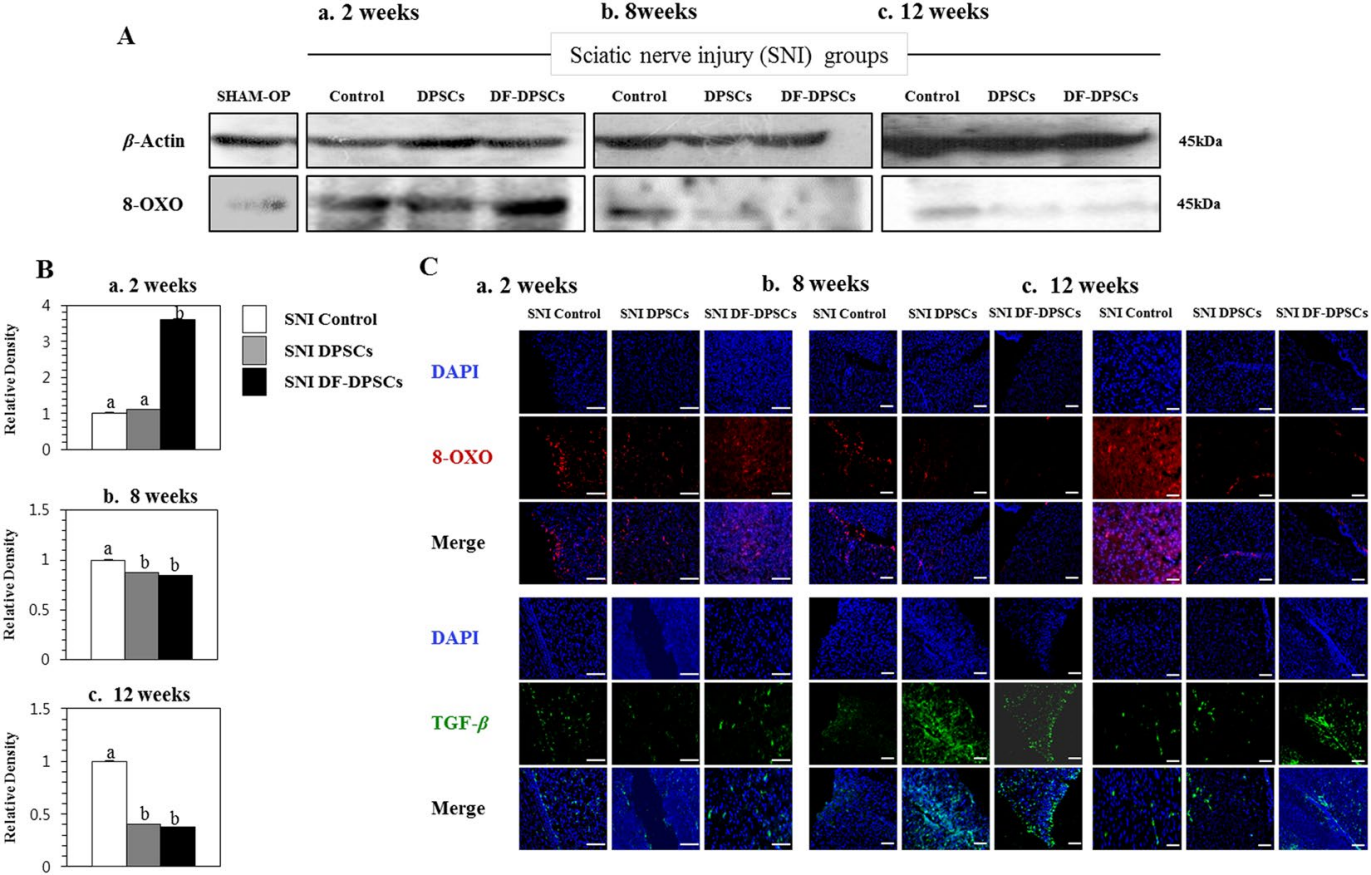
Protein expression analysis of ROS and TGF-*β* in rat brain 2, 8 and 12 weeks after surgery. Western blot analysis of 8-OXO (**A**a) 2 weeks, (**A**b) 8 weeks, and (**A**c) 12 weeks after surgery in the SNI groups. Integrated density histograms of 8-OXO after 2, 8 and 12 weeks (**B**a–c), respectively *β*-Actin was used as an internal control. The characters a, b, and c indicate significant differences (*p* < 0.05) among the SNI groups. Immunohistofluorescence analysis of 8-OXO and TGF- *β* (**C**a) 2 weeks, (**C**b) 8 weeks, and (**C**c) 12 weeks after surgery. The blue color represents nuclear staining (DAPI) while red (8-OXO) and green (TGF-*β*) represent Alexa Fluor and FITC staining of secondary antibodies, respectively. Scale bar = 100 *µ*m. SHAM OP represents the normal control. For immunohistofluorescence analysis, 10–14 *µ*m transverse sections of the forebrain region were made. Each picture contains part of the gray cortex, cerebral cortex and white matter.

ROS specific marker (8-OXO) activity was analyzed by Western blot 2, 8 and 12 weeks after surgery. After 2 weeks, SNI DF-DPSCs had a significantly higher expression of 8-OXO compared to the SNI DPSCs and control groups (Fig. 2Aa) whereas 8 and 12 weeks after surgery 8-OXO expression was low, specifically in SNI DPSCs and DF-DPSCs (Fig. 2Ab,Ac). TGF-*β* and 8-OXO expression were further analyzed by immunofluorescence, which showed an increase in TGF-*β* levels with a decrease in 8-OXO after 8 and 12 weeks in the DPSCs and DF-DPSCs groups (Fig. 2Cb,Cc), especially after 8 weeks. The expression of 8-OXO was also analyzed by Western blot in the SHAM OP group, which showed similarities with the SNI DPSCs and DF-DPSCs groups at 8 and 12 weeks after surgery. Figure Ba-c showed representative relative band density histograms.

### Protein expression of pro- and anti-inflammatory cytokines

**Figure 3:**
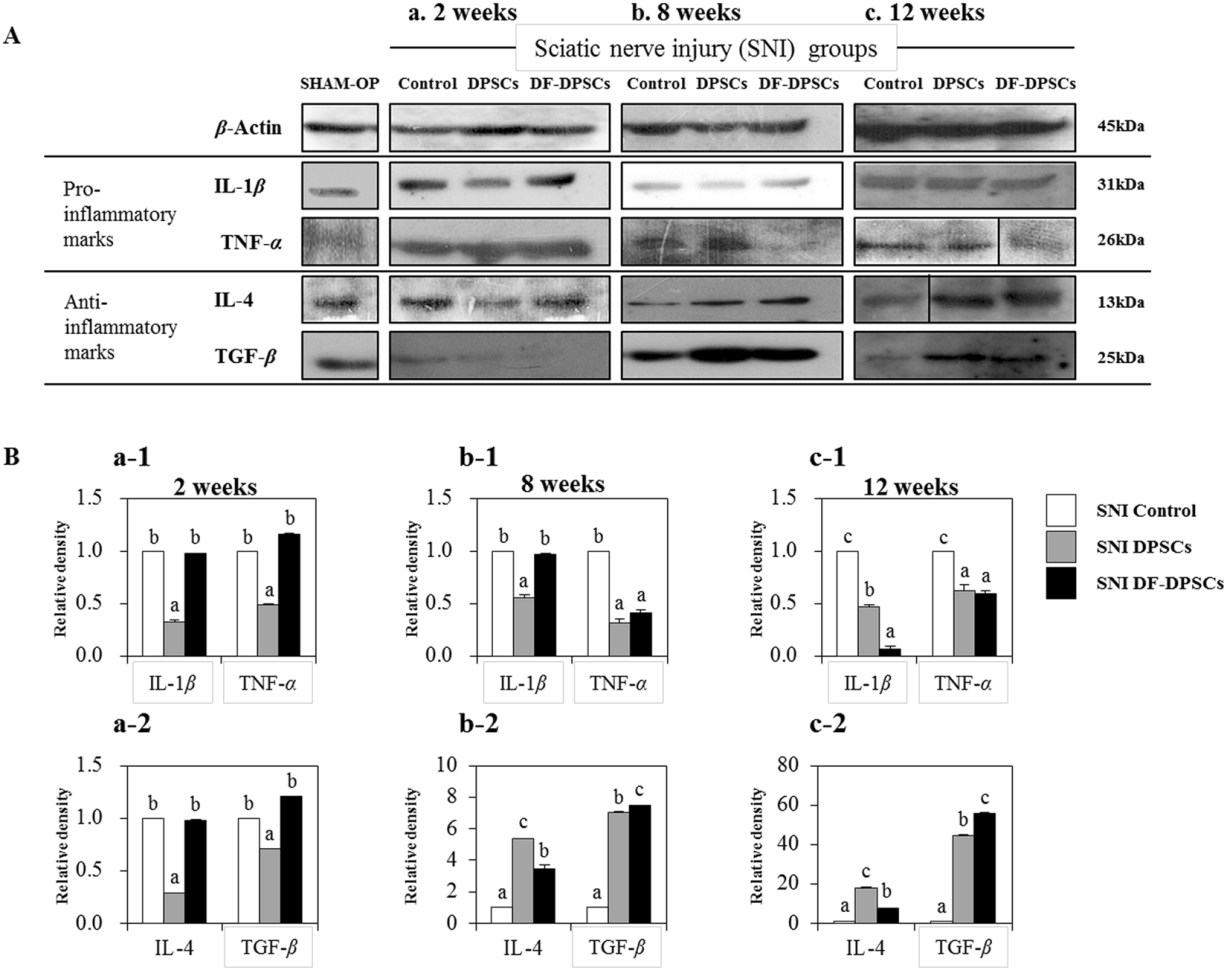
Western blot analysis of pro- (IL-1*β*, TNF-*α*) and anti-inflammatory (IL-4, TGF-*β*) cytokines in rat brain (**A**a) 2 weeks, (**A**b) 8 weeks, and (**A**c) 12 weeks after surgery. Integrated density histograms of pro- (a-1, b-1, and c-1) and anti-inflammatory (a-2, b-2, and c-2) cytokines in rat brain 2, 8 and 12 weeks after surgery, respectively. *β-*Actin was used as an internal control. The characters a, b, and c indicate significant differences (*p* < 0.05) among the SNI groups. SHAM OP represents the normal control.

Specific pro- (IL-1*β* and TNF-*α*) and anti-inflammatory cytokine (IL-4 and TGF-*β*) expression was evaluated by Western blot 2, 8 and 12 weeks post-surgery. After 2 weeks, we found low expression of pro-inflammatory (IL-1*β* and TNF-*α*) cytokines in SNI DPSCs compared to SNI DF-DPSCs and control, and this pattern of expression was maintained in anti-inflammatory cytokines (IL-4 and TGF-*β*) (Fig. 3Aa). After 8 weeks, the TNF-*α* level began to decline in SNI DF-DPSCs compared to the control group but was not significantly different between SNI DPSCs and SNI DF-DPSCs (Fig. 3Bb-1). Furthermore, IL-4 and TGF-*β* levels dramatically increased in both SNI DPSCs and SNI DF-DPSCs compared to the control group (Fig. 3Ab,Bb-2). IL-4 was higher in SNI DPSCs while TGF-*β* was higher in SNI DF-DPSCs, and this phenomenon was maintained at 12 weeks (Fig. 3Ac,Bc-2). Pro- and anti-inflammatory cytokine expression was also found in the SHAM OP group, which was consistent with the control SNI groups (Fig. 3Aa). Both pro- and anti-inflammatory cytokine expression levels in the SHAM OP group were agonistic in the SNI DPSCs and DF-DPSCs groups after 8 and 12 weeks. Figures a1, a2, b1, b2, c1 and c2 showed representative relative band density histograms.

### mRNA expression of pro- and anti-inflammatory cytokines

**Figure 4:**
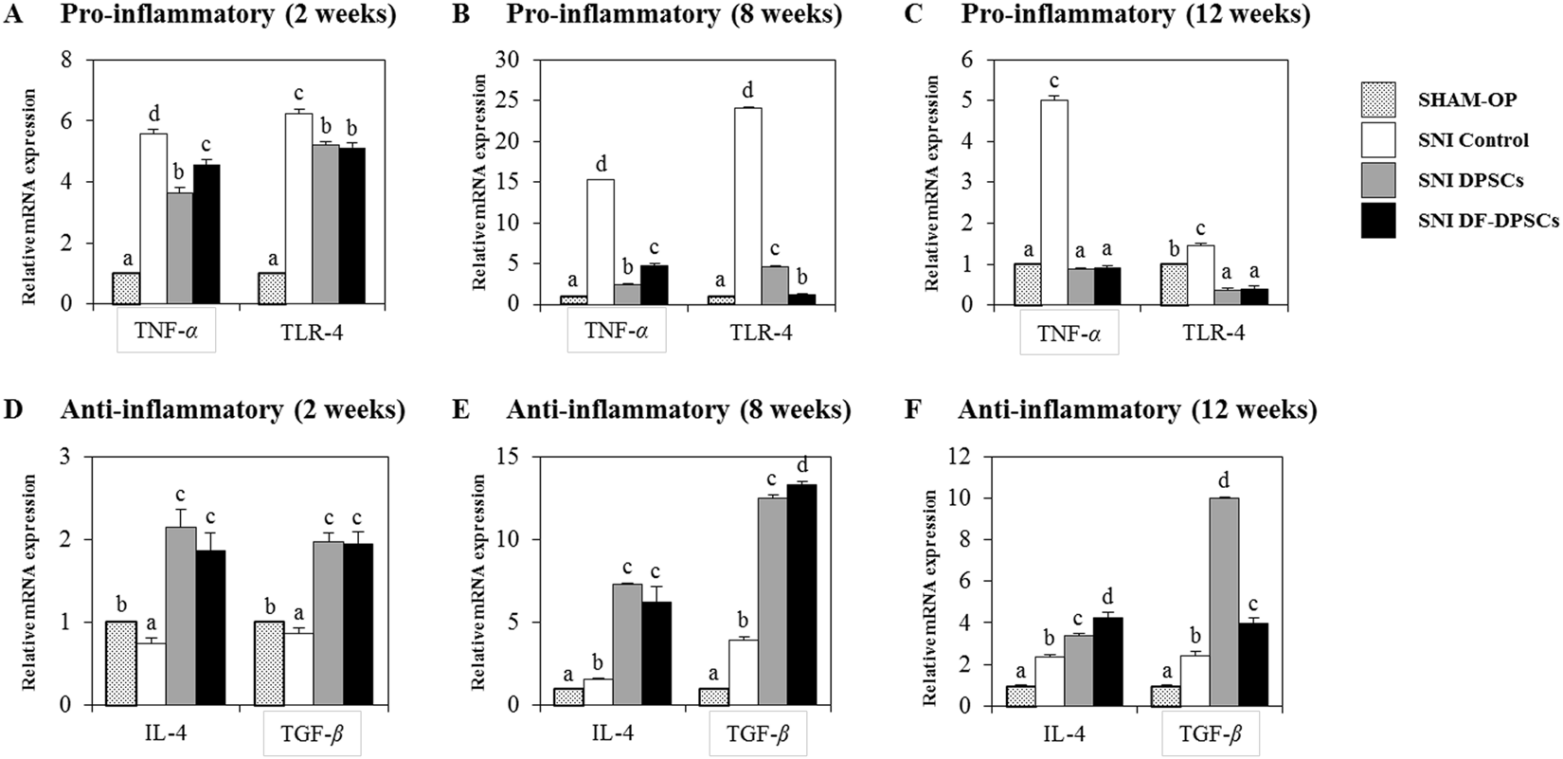
RT-qPCR analysis of inflammatory cytokines in rat brain. RT-qPCR analysis of pro- (*Tnf*-*α*, *Tlr*) and anti-inflammatory (*Il-4*, *Tgf*-*β*) cytokines in rat brain (**A**,**D**) 2 weeks, (**B**,**E**) 8 weeks, and (**C**,**F**) 12 weeks after surgery. The letters a, b, c, and d indicate significant differences (*p* < 0.05) in the expression of mRNA among the SHAM OP and SNI groups.

The expression levels of pro-inflammatory (*Tnf-α, Tlr-4*) and anti-inflammatory (*Il-4, Tgf-β*) markers were analyzed in rat brains by RT-qPCR at 2, 8 and 12 weeks after surgery relative to the SHAM OP group. At 2 weeks, the expression of *Tnf-α* (SNI control: 5.6 ± 0.145, DPSCs: 3.6 ± 0.177, DF-DPSCs: 4.5 ± 0.181) and *Tlr-4* (SNI control: 6.2 ± 0.152, DPSCs: 5.2 ± 0.119, DF-DPSCs: 5.1 ± 0.131) was higher in all three SNI groups (Fig. 4A). However, at 8 weeks, the expression significantly decreased (*p* < 0.05) in DPSCs (*Tnf-α*: 2.4 ± 0.087, *Tlr-4*: 4.5 ± 0.188) and DF-DPSCs (*Tnf-α*: 3.4 ± 0.244, *Tlr-4*: 1.2 ± 0.104) compared to controls (*Tnf-α*: 15.2 ± 0.084, *Tlr*-4: 24.0 ± 0.146) (Fig. 4B). Moreover, consistent results were found at 12 weeks in the controls (*Tnf-α*: 5.0 ± 0.108, *Tlr-4*: 1.4 ± 0.069), DPSCs (*Tnf-α*: 0.9 ± 0.03, *Tlr-4*: 0.3 ± 0.07) and DF-DPSCs (*Tnf-α*: 0.9 ± 0.051, *Tlr-4*: 0.4 ± 0.085) (Fig. 4C). In contrast, the expression of anti-inflammatory cytokines was lower at 2 weeks (*Il-4* SNI control: 0.7 ± 0.059, DPSCs: 2.8 ± 0.207, DF-DPSCs: 1.9 ± 0.216; *Tgf-β* SNI control: 0.9 ± 0.069, DPSCs: 1.9 ± 0.112, DF-DPSCs: 1.9 ± 0.145) (Fig. 4D) while significantly increased (*p *< 0.05) at 8 weeks (*Il-4* SNI control: 1.6 ± 0.090, DPSCs: 7.3 ± 0.129, DF-DPSCs: 6.4 ± 0.90; *Tgf-β* control: 3.9 ± 0.206, DPSCs: 12.2 ± 0.202, DF-DPSCs: 13.2 ± 0.253) (Fig. 4E) and 12 weeks (*Il-4* SNI control: 2.3 ± 0.133, DPSCs: 3.5 ± 0.145, DF-DPSCs: 4.9 ± 0.253; *Tgf-β* SNI control: 2.4 ± 0.0.202, DPSCs: 9.9 ± 0.061, DF-DPSCs: 4.2 ± 0.234) (Fig. 4F).

### Antagonistic mechanistic analysis of pAMPK/SIRT1 and NFkB

**Figure 5:**
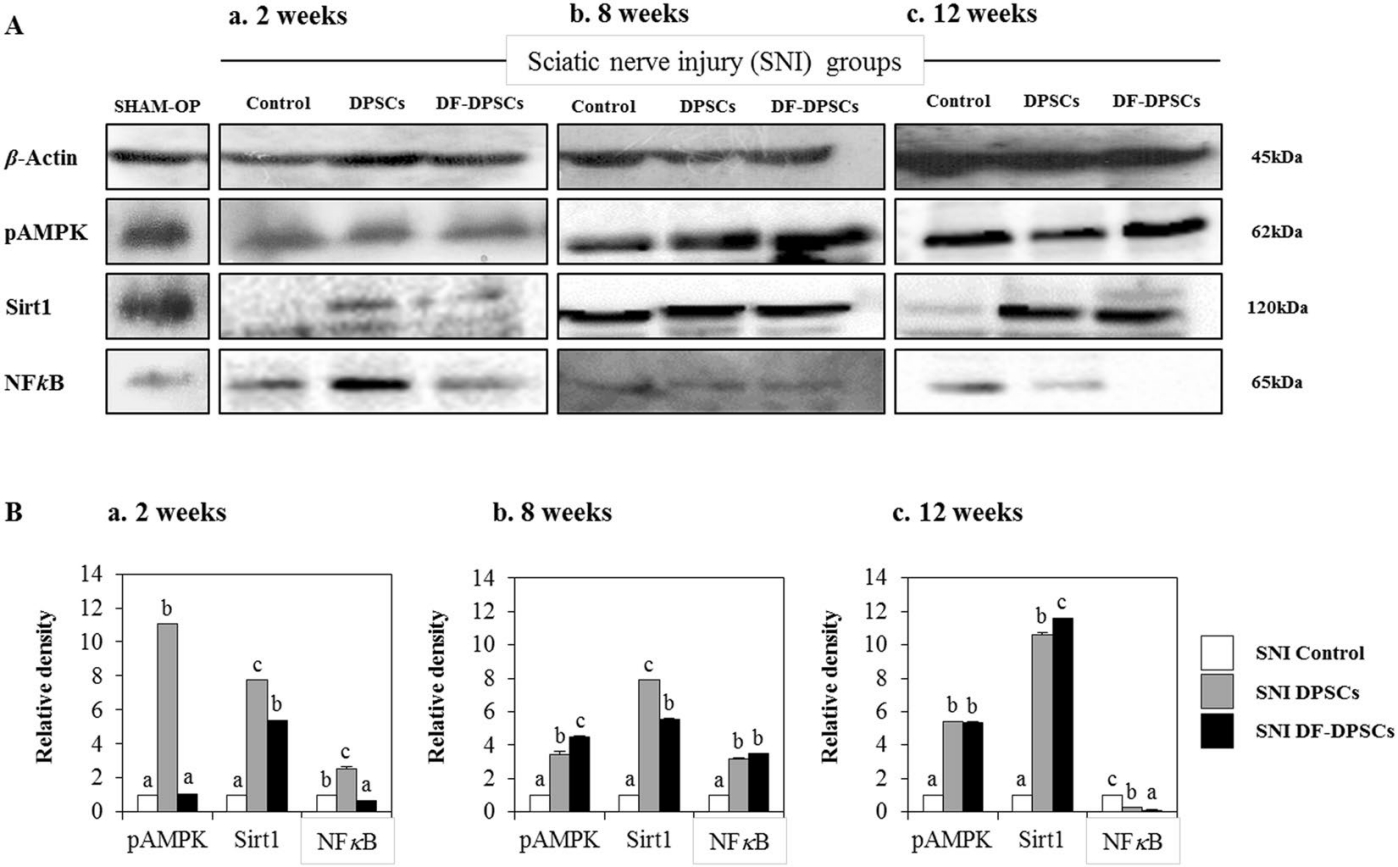
Western blot analysis of pAMPK, SIRT1 and NFkB in rat brain 2, 8 and 12 weeks after surgery. Western blot analysis of pAMPK, SIRT1 and NFkB (**A**a) 2 weeks, (**A**b) 8 weeks, and (**A**c) 12 weeks after surgery. Integrated density histograms of pAMPK, SIRT1 and NFkB after 2, 8 and 12 weeks (**B**a–c), respectively. *β*-Actin was used as an internal control. The characters a, b, c, and d indicate significant differences (*p* < 0.05) among the SNI groups. SHAM OP represents the normal control.

Expression levels of neuro pain inhibition pathway-related NFkB and pAMPK/SIRT1, as an antagonist of NFkB, were analyzed in rat brains 2, 8 and 12 weeks after surgery in all groups. After 2 weeks, we found a high expression of pAMPK, SIRT1 and NFkB in SNI DPSCs. However, SNI DF-DPSCs had higher SIRT1, low NFkB and a similar expression of pAMPK compared to the SNI control group (Fig. 5Aa). After 8 weeks, both SNI DPSCs and SNI DF-DPSCs had high expression of these three proteins compared to the control (Fig. 5Ab). Between SNI DPSCs and SNI DF-DPSCs, pAMPK was higher in SNI DF-DPSCs; SIRT1 was higher in SNI DPSCs, while NFkB was the same in the two groups (Fig. 5Bb). Moreover, after 12 weeks, SNI DPSCs and SNI DF-DPSCs had high expression of pAMPK and SIRT1 compared to SNI control. However, SIRT1 expression was higher and NFkB expression lower in SNI DF-DPSCs (Fig. 5Bc). The expression pattern of these proteins in the SHAM OP group was comparatively equal to the SNI DPSCs and DF-DPSCs groups 8 and 12 weeks after surgery. Figure Ba–c shows representative relative band density histograms.

### Immunohistofluorescence analysis of pAMPK, IL-1*β*, and NFkB in the brains of the SNI rat model

**Figure 6:**
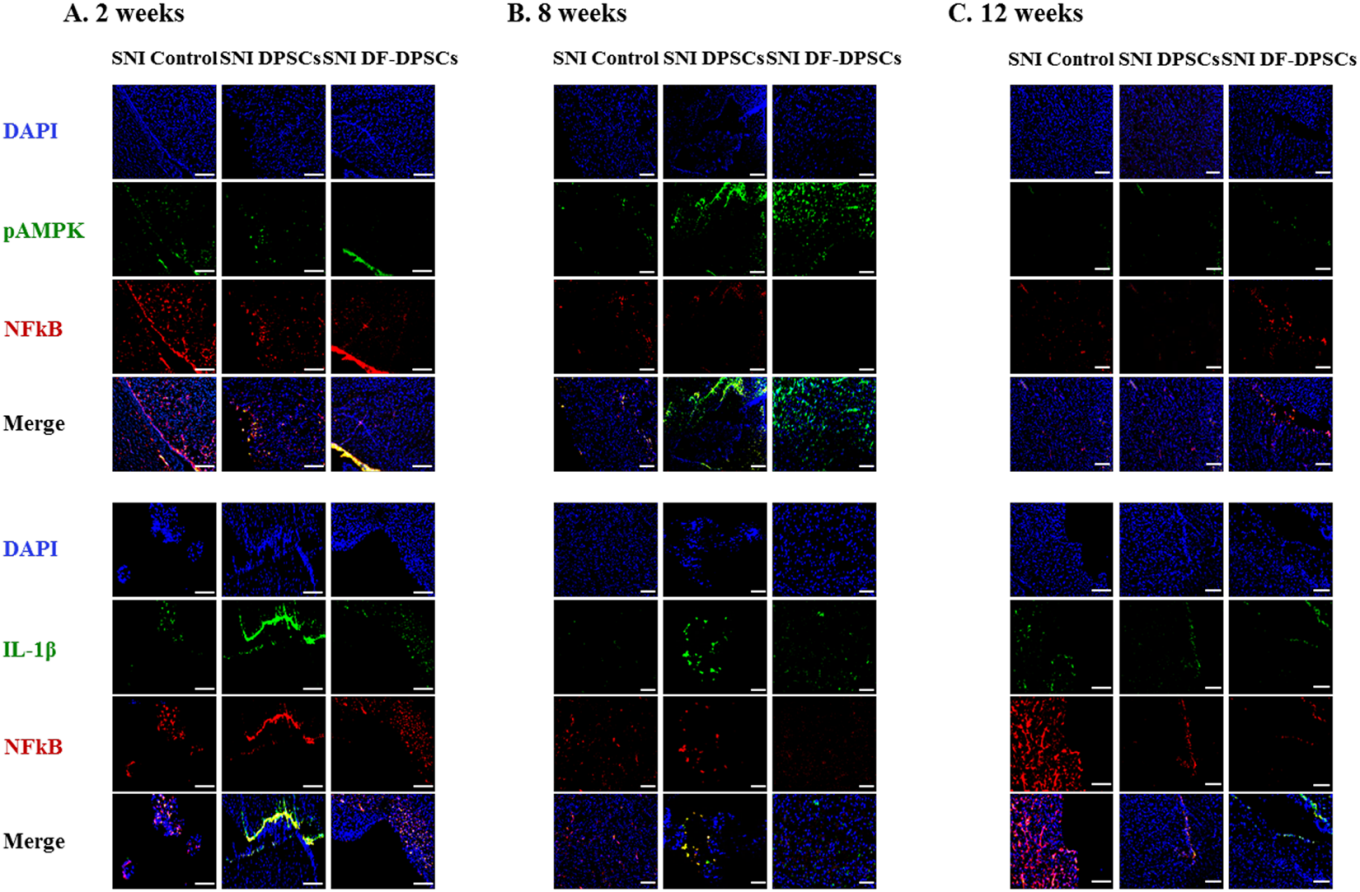
Immunohistofluorescence analysis pAMPK, IL-1*β* and NFkB proteins in rat brain 2, 8 and 12 weeks after surgery. The double immunohistofluorescence images of pAMPK/NFkB and IL-1β/NFkB in rat brain (**A**) 2 weeks, (**B**) 8 weeks, and (**C**) 12 weeks after surgery. The blue color represents nuclear staining (DAPI) while red (NFkB) and green (pAMPK and IL-1β) represent Alexa Fluor and FITC staining of secondary antibodies, respectively. Scale bar = 100 *µ*m. For immunohistofluorescence analysis, 10–14 *µ*m transverse sections of the forebrain region were made. Each picture contains part of the gray cortex, cerebral cortex and white matter.

The expression of certain specific proteins was analyzed by immunohistofluorescence. We performed double antibody staining of rat brain sections at 2, 8 and 12 weeks for pAMPK/NFkB and IL-1*β*/NFkB and found a lower expression of pAMPK and a higher expression of NFkB in all of the SNI groups at 2 weeks (Fig. 6A). Similarly, the expression of IL-1*β* was relatively higher in controls compared to DF-DPSCs (Fig. 6A). However, 8 weeks after surgery, there was a higher expression of pAMPK in DPSCs and DF-DPSCs compared to the control and a lower expression of NFkB (Fig. 6B). Similarly, IL-1*β* expression was significantly down-regulated with NFkB (Fig. 6B). Similar results were found in the 12-week samples, showing significantly higher expression of pAMPK (Fig. 6C) and lower expression of IL-1*β* and NFkB (Fig. 6C).

## Discussion

Previously, we reported in detail about sciatic nerve regeneration using DPSCs and DF-DPSCs^4^. In this study, we analyzed the relieving effect of dental pulp stem cells on supraspinal neuro-inflammation caused by inflammatory mediators, which are increased due to peripheral neuropathy, followed by higher ROS and NFkB activity and homeostasis dysregulation.

Microglia in the nervous system serve as resident immune cells and are sensitive sensors of certain events that occur in their immediate environment^26^. In a healthy brain, microglias in close contact with neurons are in an inactive form^27,28^. Stress or damaged neurons have the potential to express specific factors that stimulate the production of pro-inflammatory cytokines by microglia^26^. In our study, we observed similar phenomena and found higher expression of a microglia marker (GFAP) at the onset of nerve injury. The inflammatory responses were initiated by pattern recognition receptors (PRRs), i.e., toll-like receptors (TLRs), advanced glycation end products receptors (RAGE) and scavenger receptors^26^. Ligation of PPRs activates signal transduction pathways and regulates diverse transcriptional and post-transcriptional molecules. These molecules include members of NFkB, activator protein 1 (AP-1), and interferon regulator factor families, which modulate pro-inflammatory genes encoding chemokines, cytokines, enzymes and other essential molecules^27^. Furthermore, in chronic inflammatory diseases, microglial activation leads to release of stress-associated molecules and inflammatory cytokines^25^. The stress-associated molecules are reactive oxygen species (ROS) and reactive nitrogen species (RNS). The oxidative stress in neural tissues causes disrupted signaling and homeostasis and is responsible for numerous events ranging from protein misfolding to the death of neurons^29,30^. The higher expression levels of ROS and RNS also activate downstream NFkB, which in turn initiates pro-inflammatory factor transcription. We observed that at the onset of sciatic nerve injury (2 weeks), microglia activation by Iba1 expression induced higher ROS production and subsequently activated downstream NFkB, resulting in a higher pro-inflammatory response. In addition to microglial and ROS activation, depressive behavior also contributes to a strong inflammatory response with higher expression of TNF and IL-6^18,19^. We observed a similar situation and found that mRNA expression of pro-inflammatory markers, including TNF-*α* and TLR-4, was significantly higher in the SNI control, SNI DPSCs and DF-DPSCs groups. However, the expression of IL-1*β* and TNF-*α* gradually decreased at 8 weeks and 12 weeks in the SNI DPSCs and DF-DPSCs groups compared to the SNI control. Alternatively, the expression of anti-inflammatory cytokines, including IL-4 and TGF-*β*, was significantly lower 2 weeks after surgery, while the expression was higher in the 8 and 12 week samples, especially in the SNI DPSCs and DF-DPSCs groups. Our results are inconsistent with previous findings^31^. Previously, patients with neuropathy and depression had higher levels of pro-inflammatory cytokines such as TNF-*α*^20^. TLR signaling induces a higher pro-inflammatory response in microglia, which further secrete IL-12, TNF-*α*, IL-1*β* and IL-6^32^. Our results are consistent with this study, and we found a higher expression of TNF-*α*, IL-1*β* and TLR-4 after 2 weeks of surgery. Our findings suggest that at the onset of sciatic nerve injury, the rats were depressed and stressed due to a high level of neuropathic pain, which leads to microglial activation and higher ROS activity. However, as the DPSCs and DF-DPSCs supported nerve regeneration, the body was less stressed, leading to a higher expression of anti-inflammatory cytokines and a lower expression of pro-inflammatory cytokines at 8 weeks and 12 weeks after surgery in the two cell-transplanted groups. Furthermore, from the above results, we found a slight difference in the effect of SNI DPSCs and SNI DF-DPSCs after a two-week interval. The expression patterns of IL-4, IL-1*β* and Iba1 were slightly higher in SNI DF-DPSCs compared to DPSCs. This early effect of DPSCs is a well-known characteristic of MSCs where they migrate to the site of injury and initiate immune and inflammatory responses through several paracrine mechanisms^1,2^. Here, the early response (2 weeks) of DPSCs on SNI might be due to the homing and immunomodulatory effect of these cells. However, in the fully differentiated form, DF-DPSCs had no effects or minor effects on these proteins in the early stages. Furthermore, in SNI, the microglia and astrocyte activation are well-documented with the pain level and rapid regeneration, respectively. Therefore, we hypothesized that at the early stages (2 weeks), the lower activity of microglia (Iba1) and higher activation of astroglia (GFAP) revealed a lower pain induction and rapid regeneration potential of DPSCs. However, further in-depth studies are required to fully elucidate this hypothesis.

NFkB is a transcription factor involved in the transcription of genes that encode pro-inflammatory cytokines, and its role in pain facilitation after nerve injury is well-documented^33^. We observed that a higher expression of Iba1 increased ROS levels, which led to NFkB activation at the onset of nerve injury (2 weeks) via translocation into the nucleus to initiate the transcription of pro-inflammatory cytokines while down regulating anti-inflammatory factors. Our results further revealed that 8 and 12 weeks post-surgery, the expression of GFAP and Iba1 were down-regulated, which might be due to regeneration of the sciatic nerve, which relieves neuropathic pain. The down-regulation of Iba1 decreases ROS activity, which in turn inhibits NFkB activation and leads to lower expression of pro-inflammatory cytokines and a higher expression of anti-inflammatory cytokines 8 and 12 weeks after surgery. AMP-activated protein kinase (AMPK) is the main homeostasis regulator and functions as an energy sensor^34^. AMPK plays an important role in neuronal function, plasticity and neurodegeneration^35^. SIRT1, a class III histone deacetylase downstream of AMPK^36^, is involved in many pathophysiological conditions, including diabetes and neurodegeneration^37,38^. Antagonistic characteristics of SIRT1 with NFkB have been previously reported^39^. AMPK activation can suppress NFkB and vice versa. Here, we reported that up-regulation of ROS subsequently led to NFkB activation, which in turn down-regulated pAMPK/SIRT1 activity at the onset of sciatic nerve injury (2 weeks). However, after 12 weeks, down regulation of NFkB results in up-regulation of pAMPK/SIRT1, which is inconsistent with crosstalk previously reported. Here, we again found an exception with DPSCs, which have a higher expression of pAMPK and SIRT1 at 2 weeks. This differential behavior of DPSCs has been discussed earlier in detail. In this study, we found that the SNI DPSCs and DF-DPSCs groups showed improved neuro-inflammation via expression of a specific set of inflammatory activators and inhibitors at different time intervals. These observations suggested that DPSCs (more actively) and DF-DPSCs can efficiently counterbalance peripheral nerve-induced supraspinal neuro-inflammation.

## Conclusion

The inflammation caused in the rat brain is due to peripheral neuropathy, which in turn initiates the accumulation of Iba1 and ROS and the secretion of inflammatory cytokines. At the onset of sciatic nerve injury (2 weeks), there was higher ROS activity with higher expression of GFAP and NFkB and lower expression of pAMPK/SIRT1, which leads to higher expression of pro-inflammatory cytokines. In contrast, at 8 and 12 weeks after surgery, we observed low expression of Iba1, ROS, GFAP and NFkB and higher expression of pAMPK/SIRT1 followed by lower expression of pro-inflammatory cytokines and higher expression of anti-inflammatory cytokines in the SNI DPSCs and SNI DF-DPSCs groups, which was significantly different from the SNI control. From these observations, we hypothesized that at 8 weeks and 12 weeks, the DPSCs and DF-DPSCs recovered the damaged sciatic nerve, which in turn relieved the rats’ neuropathic pain, leading to less stress, which finally reduced the supraspinal neuro-inflammation from the oxidative stress and homeostatic dysregulation after sciatic nerve injury. Furthermore, we observed an early effect (at 2 weeks) of DPSCs in reducing microglial activation, down-regulating pro-inflammatory markers and up-regulating pAMPK/SIRT1. This phenomenon suggests the homing and immunomodulatory effects as well as rapid regeneration and pain-relieving effects of MSCs, which might be not be present in differentiated DF-DPSCs. However, further studies are required to answer these questions. Finally, we conclude that DPSCs responded early and more efficiently than DF-DPSCs in relieving neuropathic pain and counterbalancing neuro-inflammation, which is consistent with our previous report. This study will help to provide further insight into the treatment of sciatic nerve injury and neuro-inflammation replacing autologous nerve grafts.

## Materials and Methods

### Animals

A total of 36 (3 rats in each group, in triplicate) adult female Sprague-Dawley rats (8 weeks old) (Charles River, Orient Bio Inc., Sungnam, Korea) were used for all surgical procedures. All animal experiments using rats were approved by the Animal Center for Medical Experimentation at Gyeongsang National University, Korea. Furthermore, all methods were performed in accordance with the relevant guidelines and regulations under approved medical guidelines (GNU IRB-2012-09-004) at Gyeongsang National University hospital.

### Isolation and differentiation of dental pulp stem cells (DPSCs)

MSCs were derived from dental pulp stem cells (DPSCs) and differentiated into neuronal cells (DF-DPSCs), as previously reported^3^.

### Peripheral nerve injury (PNI) rat model preparation and cell transplantation

Surgeries were performed to prepare the neurotmesis rat model as previously described^4^. Briefly, the rats were placed under general anesthesia with a subcutaneous injection of 0.5 μL/g of tiletamine-zolazepam (Zoletil®) and 0.5 µL/g xylazine (Rompun, Bayer Korea Ltd., Seoul, Korea). The sciatic nerve injury (SNI) model was constructed as follows. Briefly, the left sciatic nerve was exposed through a gluteal muscle incision. After exposure of the sciatic nerve, a 7–8 mm section of sciatic nerve was removed along the longitudinal axis. A conduit made from Lyoplant membrane was sutured to both the ends of the injured sciatic nerve. A total of 1 × 10^6^/20 µL pKh26 (Sigma PKH26GL, St. Louis, USA) labeled DPSCs and DF-DPSCs were transplanted into the conduit with 20 µL of Greenplast® fibrin glue (Green cross, Seoul, South Korea). The animals were divided into SHAM OP (SHAM operated) and three SNI groups as follows: (1) Control group: animals transplanted with Dulbecco’s phosphate buffer saline (DPBS) instead of stem cells, (2) DPSCs group: animals transplanted with dental pulp stem cells, (3) DF-DPSCs: animals transplanted with dental pulp differentiated neuronal cells. The SHAM OP group was used after pseudo surgery where only an incision on the skin and upper muscles was made while the sciatic nerve was keep intact.

### Real time quantitative PCR (RT-qPCR) analysis

Pro- and anti-inflammatory specific marker expression was assessed by RT-qPCR (Table 1). After specific intervals (SNI groups: 2, 4, 8 weeks), the animals were euthanized, and whole forebrain samples were collected from the SHAM OP and all SNI groups. RNA extraction, cDNA synthesis and RT-qPCR were performed as previously reported^3^. Briefly, total RNA was isolated from the whole forebrain using the RNeasy mini kit (Qiagen, CA, USA) and quantified with a Nanodrop (Optizen NanoQ). Complementary DNA (cDNA) was synthesized from total purified RNA (2 µg) using an Omniscript reverse transcription kit (Qiagen) with 10 µM Oligo(dT) primer at 37 °C for 1 h. cDNA samples were diluted to a uniform concentration of 50 ng/µg.

**Table 1:** List of primers used in RT-qPCR.

No.	Gene (Rat)	Sequence (5′-3′)	Accession No.	Size
1	TLR-4	F: GATTGCTCAGACATGGCAGTTTC R: CACTCGAGGTAGGTGTTTCTGCTAA	NM_019178.1	**135**
2	TNF-α	1F: ACTCCCAGAAAAGCAAGCAA 1R: CGAGCAGGAATGAGAAGAGG	NM_012675.3	**211**
3	IL-4	F: AGGGTGCTTCGCAAATTTTA R: CAGTGTTGTGAGCGTGGACT	NM_201270.1	**156**
4	TGF-β1	1F: GGAGAGAAACCCTCTGAAAA 1R: CTTTCAAGAGTTTGAAGCTGAG	NC_005100.4	**158**

The RT-qPCR reaction was performed using Rotor Gene Q (Qiagen) with 50 ng of cDNA quantified with Rotor-Gene^TM^ 2X SYBR® Green mix (Qiagen) supplemented with 10 µM of specific primer sets (Table-2). The RT-qPCR reaction was performed with an initial denaturation at 95 °C for 10 minutes, followed by 40 cycles of PCR at 95 °C for 10 seconds, 60 °C for 6 seconds and 72 °C for 6 seconds. The melting curves, amplification curves and cycle threshold values (Ct) were determined by using Rotor-Gene Q series software (Qiagen). Data analysis was performed using the *ΔΔCT* method. All samples were run in triplicate, and *Gapdh* was used as an internal control.

### Western blot analysis

Western blot analysis was performed as previously reported^40^. Briefly, animals were euthanized after surgeries (2, 8, 12 weeks), and brain samples (whole forebrain containing hippocampus and thalamus) were carefully collected from all SNI groups (control, DPSCs. DF-DPSCs) and normal controls (Sham OP) and placed on dry ice to freeze the tissue. Protein lysate was prepared from the whole forebrain of SHAM OP and all of the SNI groups using RIPA buffer (PIERCE, Rockford, IL, USA) containing protease inhibitor and was further quantified using a BCA protein assay kit (PIERCE). Each protein sample (20 µg) was separated using 8–12% sodium dodecyl sulfate polyacrylamide gel electrophoresis (SDS-PAGE) for 3 h at 100 V and transferred to polyvinylidene difluoride membrane (PVDF, Bio-Rad, Hercules, CA, USA) overnight at 30 V. Then, the membranes were blocked with 5% bovine serum albumin (BSA) in Tris buffered saline (1 × -TBS) for 1 h at room temperature, followed by washing in 0.1% Tris buffered saline-Tween (TBST). The membranes were incubated with the following primary antibodies: goat anti-IL-1*β* (sc-1251, 1:1000), mouse anti-TNF-*α* (sc-33639, 1:1000), mouse anti-IL-4 (sc-53084, 1:1000), mouse anti-TGF-*β* (sc-80346, 1:1000), rabbit anti-Iba1 (sc-98468, 1:1000), mouse anti-GFAP (sc-33673, 1:1000), rabbit anti-*β*-actin (cell signaling, 4967, 1:5000), mouse anti-8OXO (sc-130914, 1:1000), mouse anti-NFkB (sc-136548, 1:1000), rabbit anti-SIRT1 (sc-15404, 1:1000), and rabbit anti-pAMPK (sc-33524, 1:1000) overnight at 4 °C. After washing three times with 0.1% TBST, the membranes were incubated with horseradish peroxidase (HRP)-conjugated goat anti-mouse (1:1000, Santa Cruz), rabbit anti-goat (1:1000, Santa Cruz) and goat anti-rabbit (1:1000, Santa Cruz) secondary antibodies for 1 h at room temperature. Immunoreactivity was detected by enhanced chemiluminescence (ECL; SuperSignal® West Pico chemiluminescent substrate, PEIRCE, Rockford, IL, USA). The membranes were then exposed to X-ray film in the dark. The X-ray films were then scanned, and optical densities of the bands were analyzed through densitometry using the computer-based ImageJ software (ImageJ Java 1.6.0_24 NIH, USA). Optical densities were first relatively measured with *β*-actin followed by the control group. The final density graph represents relative density to the control.

### Immunohistofluorescence analysis of rat brain tissues

Brain samples were collected from rats at 2, 8 and 12 weeks post-surgical interval as reported previously^40^. Briefly, the animals were subjected to transcardial perfusion with phosphate buffer saline followed by 4% ice-cold paraformaldehyde. After fixation, the tissues were transferred to 20% sucrose for 48 hours. The tissues were then frozen in OCT compound (Tissue-Tek O.C.T. Compound Medium, Sakura Finetek USA, Inc., Torrance, CA, USA) and transverse sectioned into 10–14 *µ*m sections from the forebrain regions containing part of the gray cortex, cerebral cortex and white matter using a cryotome (CM 3050 S Cryostat, Leica, Wetzlar, Germany). The sections were thaw mounted in probe-On positively charged slides (Thermo Fisher Scientific Inc., Waltham, MA, USA). Immunofluorescence staining was performed as previously reported^40^. Briefly, brain section slides were washed with 1X PBS and incubated with proteinase K for 10 min at room temperature. The slides were then blocked with normal bovine serum and primary antibodies were applied (mentioned in Western blot section) overnight at 4 °C. After primary antibody treatment, the slides were washed with DPBS and incubated with FITC or Alexa Fluor conjugated secondary antibodies (goat anti-rabbit sc-2012; goat anti-mouse sc-362277) for 1 h. For nuclear staining, the cells were treated with 1 µg/ml 4′,6-diamidino-2-phenylindole (DAPI) for 5 min at room temperature (rt). Finally, the slides were mounted with VECTASHIELD Antifade Mounting Medium (H-1000 Vector labs, USA) and images were taken using a confocal microscope (FluoView 1000, Olympus, Japan).

### Statistical analysis

Data were analyzed by one-way ANOVA followed by Tukey’s test using SPSS 21.0 (SPSS Inc., Chicago, Il, USA). Data were expressed as the means ± standard error of the mean (SE). Differences were considered significant at *p* < 0.05. All experiments were performed in triplicate.

## Electronic supplementary material

Supplementaryfile
