# Supplementary material for: Dental pulp-derived stem cells can counterbalance peripheral nerve injury-induced oxidative stress and supraspinal neuro-inflammation in rat brain
Source: Sci Rep. 2018 Oct 25;8:15795. doi: 10.1038/s41598-018-34151-x (PMC6202384; doi:10.1038/s41598-018-34151-x)
Supplement: Supplementary file 1 — Supplementaryfile [file 41598_2018_34151_MOESM1_ESM.pdf]

Supplementary Data

Figure 1

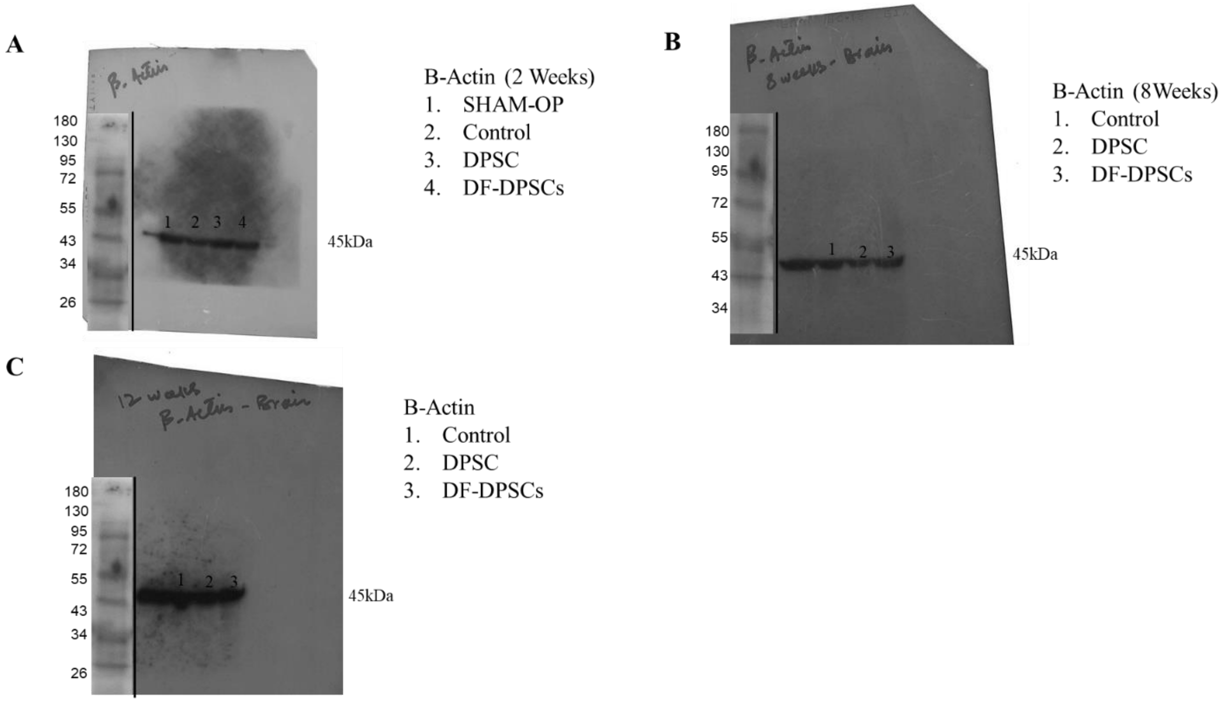

Figure 1. The whole gels immunoblot of β-Actin (45kDa) protein expressions at (A) 2 (B) 8 and (C) 12 weeks after surgery in rat brain. The bands were cropped at the exact kDa and are shown in the main manuscript and quantified using ImageJ software, and the differences are represented by a histogram.

Figure 2

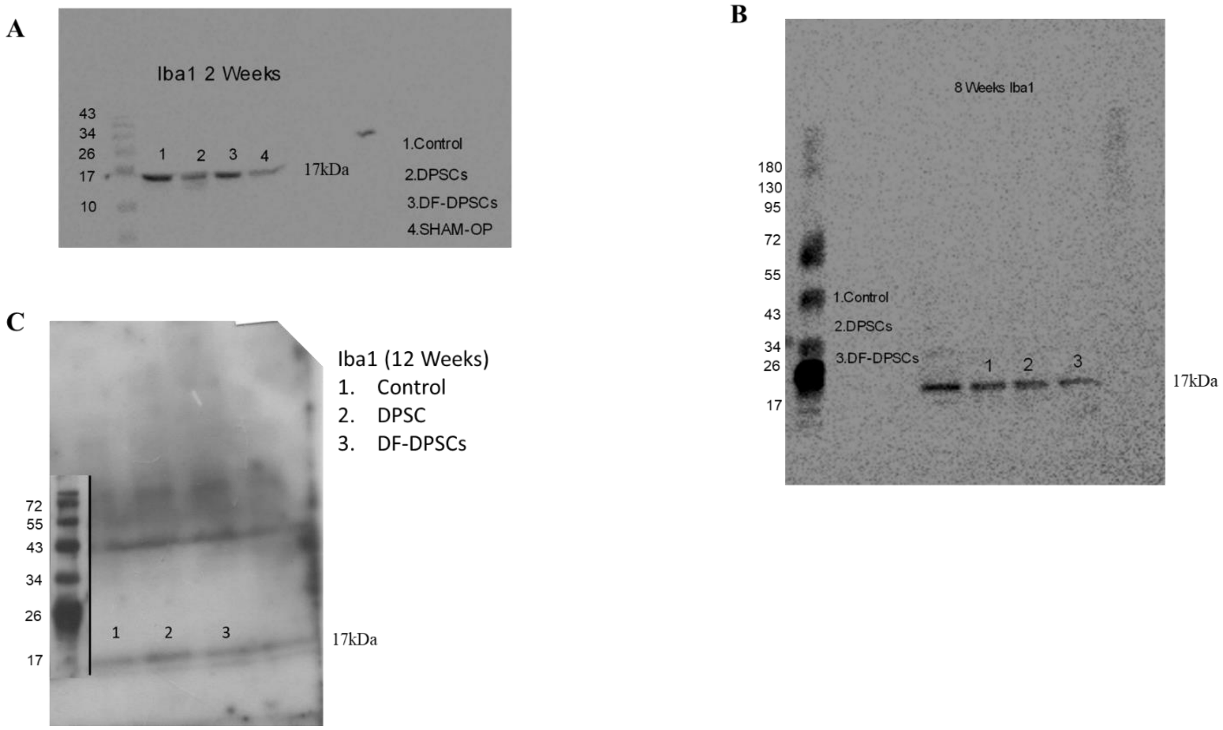

Figure 2. The whole gels immunoblot of Iba1 (17kDa) protein expressions at (A) 2 (B) 8 and (c) 12 weeks after surgery in rat brain. The bands were cropped at the exact kDa and are shown in the main manuscript and quantified using ImageJ software, and the differences are represented by a histogram.

Figure 3

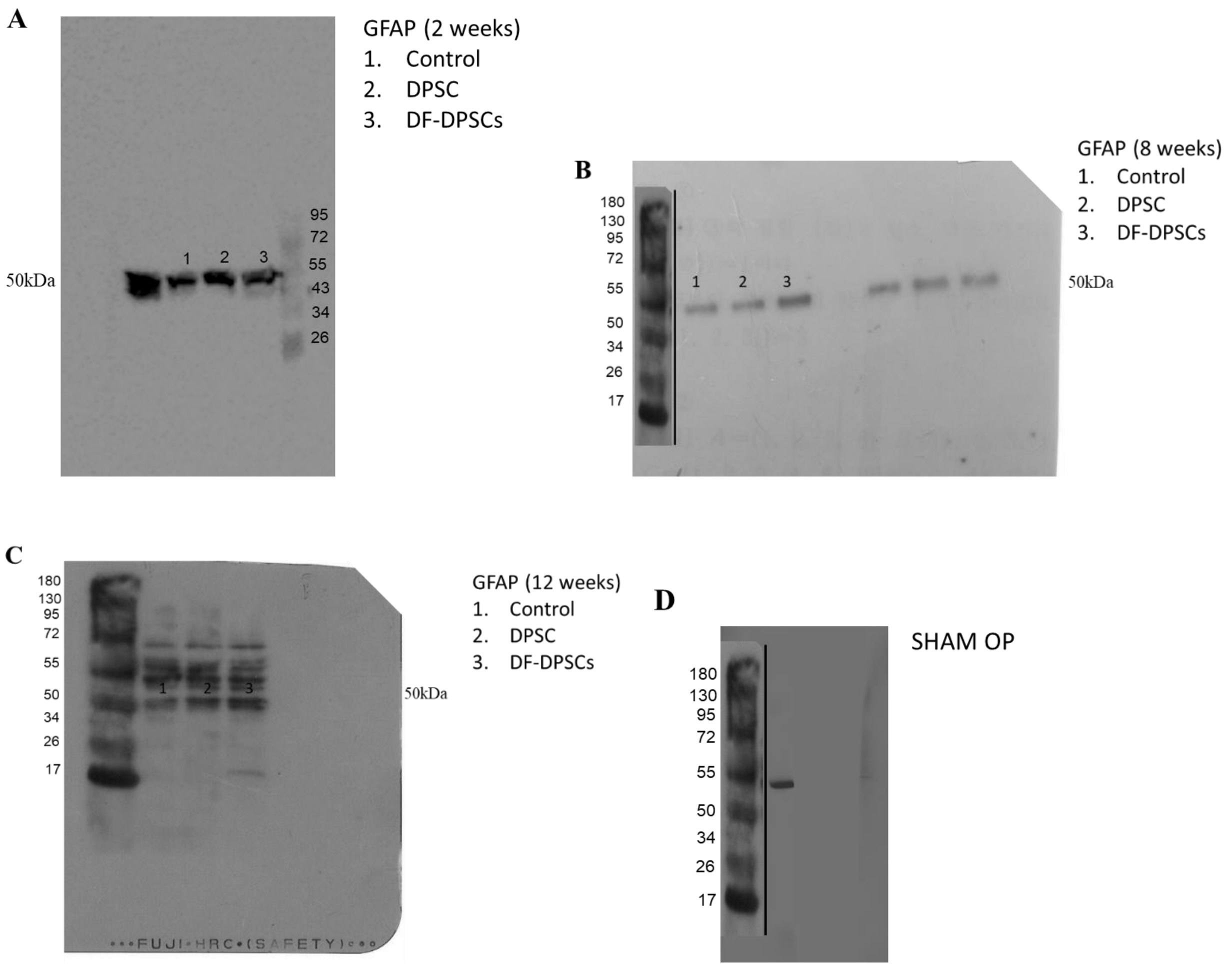

Figure 3. The whole gels immunoblot of GFAP (50kDa) protein expressions at (A) 2 (B) 8 and (c) 12 weeks after surgery in rat brain. The bands were cropped at the exact kDa and are shown in the main manuscript and quantified using ImageJ software, and the differences are represented by a histogram. (D) represents expression of GFAP in SHAM OP.

Figure 4

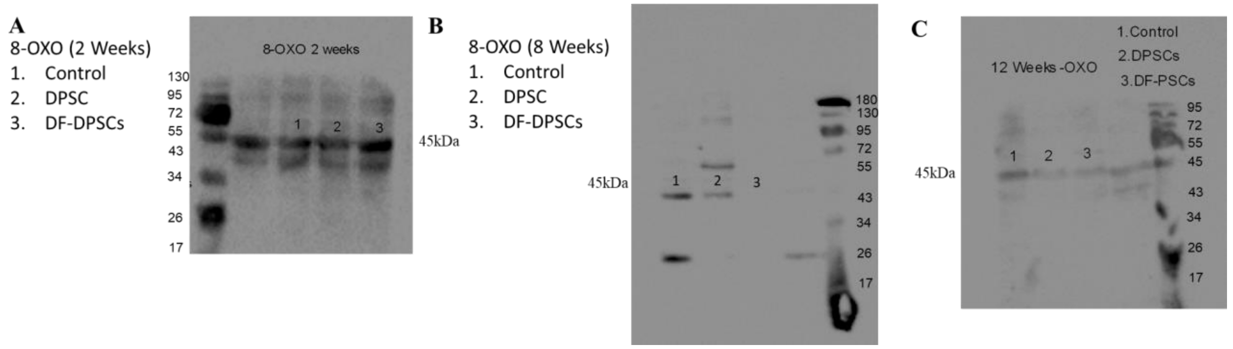

Figure 4. The whole gels immunoblot of 8-OXO (45kDa) protein expressions at (A) 2 (B) 8 and (C) 12 weeks after surgery in rat brain. The bands were cropped at the exact kDa and are shown in the main manuscript and quantified using ImageJ software, and the differences are represented by a histogram.

Figure 5

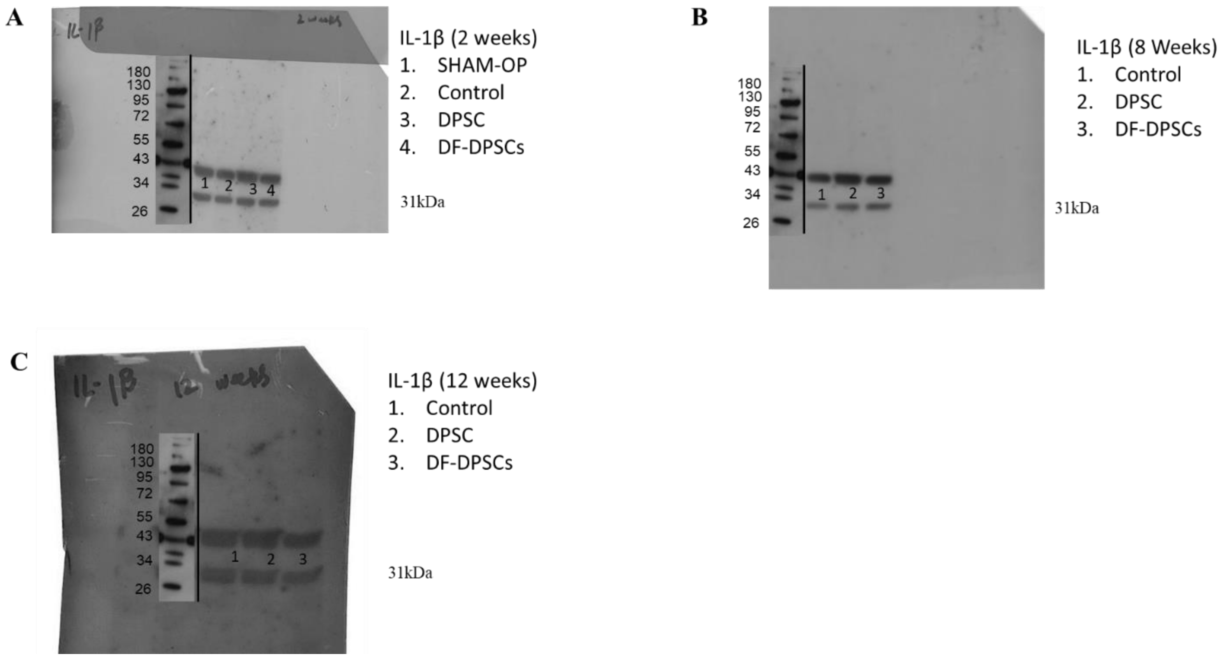

Figure 5. The whole gels immunoblot of IL-1β (31kDa) protein expressions at (A) 2 (B) 8 and (C) 12 weeks after surgery in rat brain. The bands were cropped at the exact kDa and are shown in the main manuscript and quantified using ImageJ software, and the differences are represented by a histogram.

Figure 6

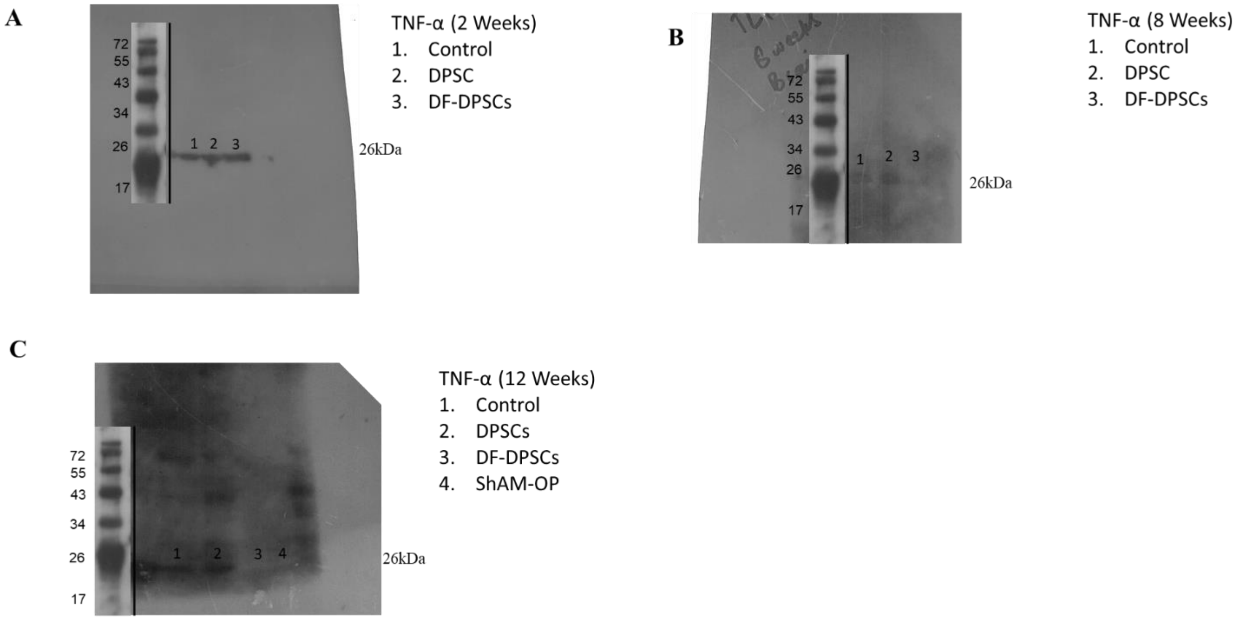

Figure 6. The whole gels immunoblot of TNF-α (26kDa) protein expressions at (A) 2 (B) 8 and (C) 12 weeks after surgery in rat brain. The bands were cropped at the exact kDa and are shown in the main manuscript and quantified using ImageJ software, and the differences are represented by a histogram.

Figure 7

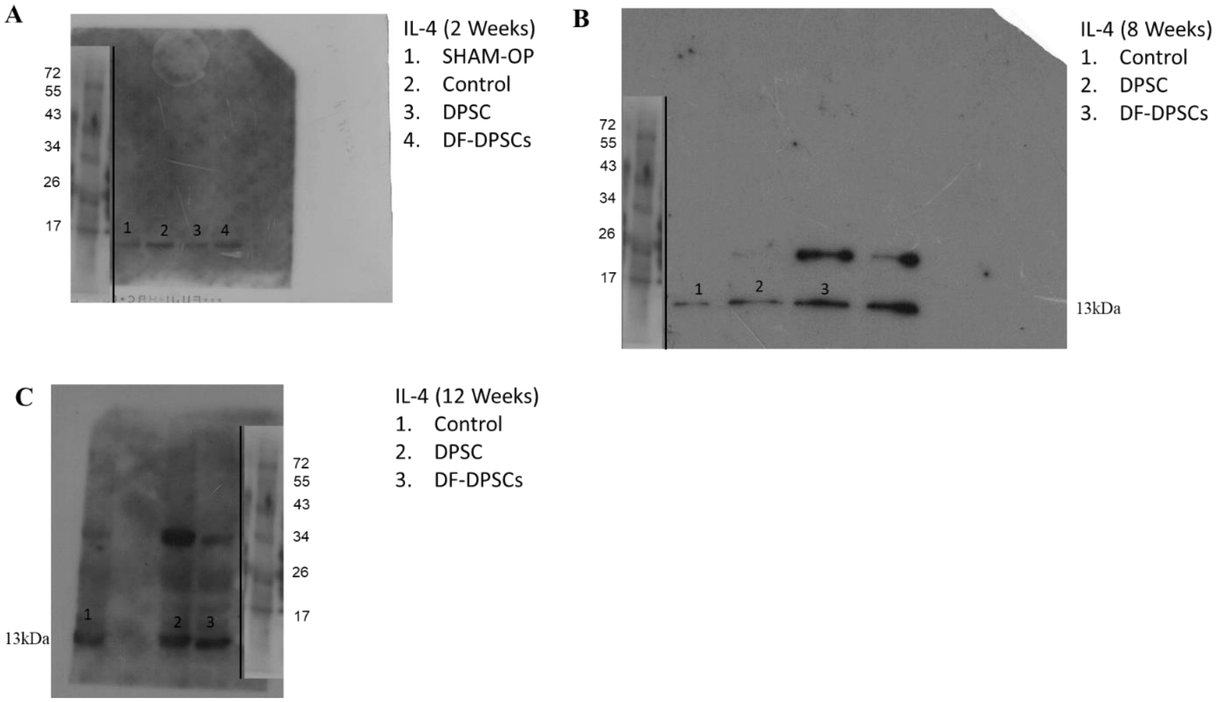

Figure 7. The whole gels immunoblot of IL-4 (13kDa) protein expressions at (A) 2 (B) 8 and (C) 12 weeks after surgery in rat brain. The bands were cropped at the exact kDa and are shown in the main manuscript and quantified using ImageJ software, and the differences are represented by a histogram.

Figure 8

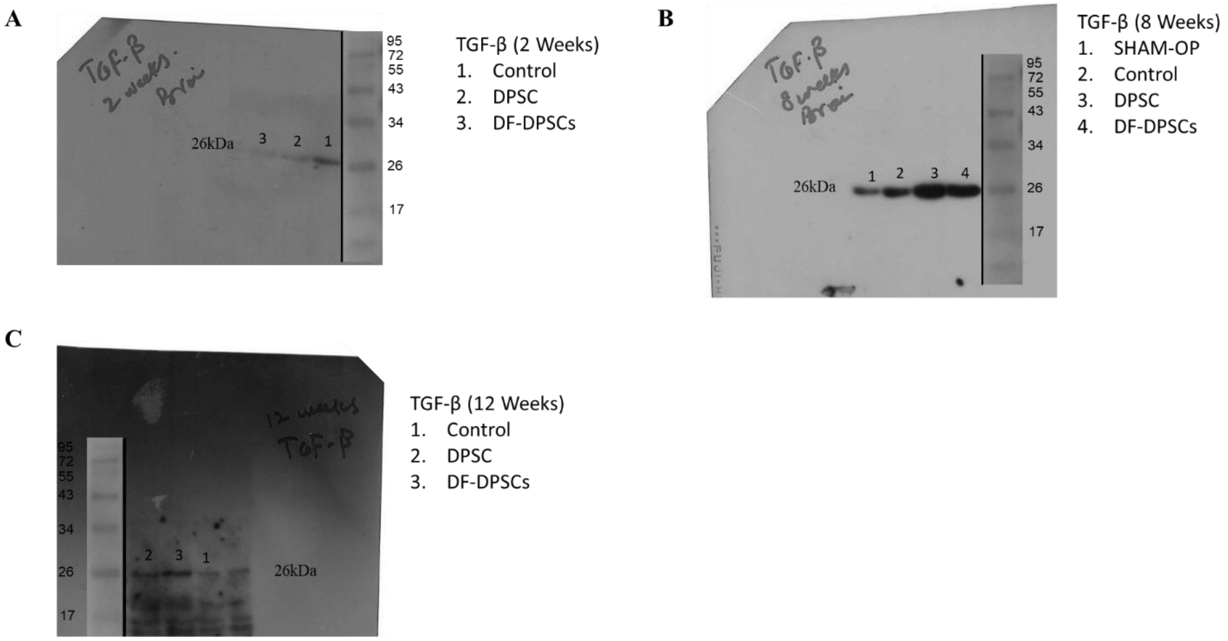

Figure 8. The whole gels immunoblot of TGF-β (26kDa) protein expressions at (A) 2 (B) 8 and (C) 12 weeks after surgery in rat brain. The bands were cropped at the exact kDa and are shown in the main manuscript and quantified using ImageJ software, and the differences are represented by a histogram.

Figure 9

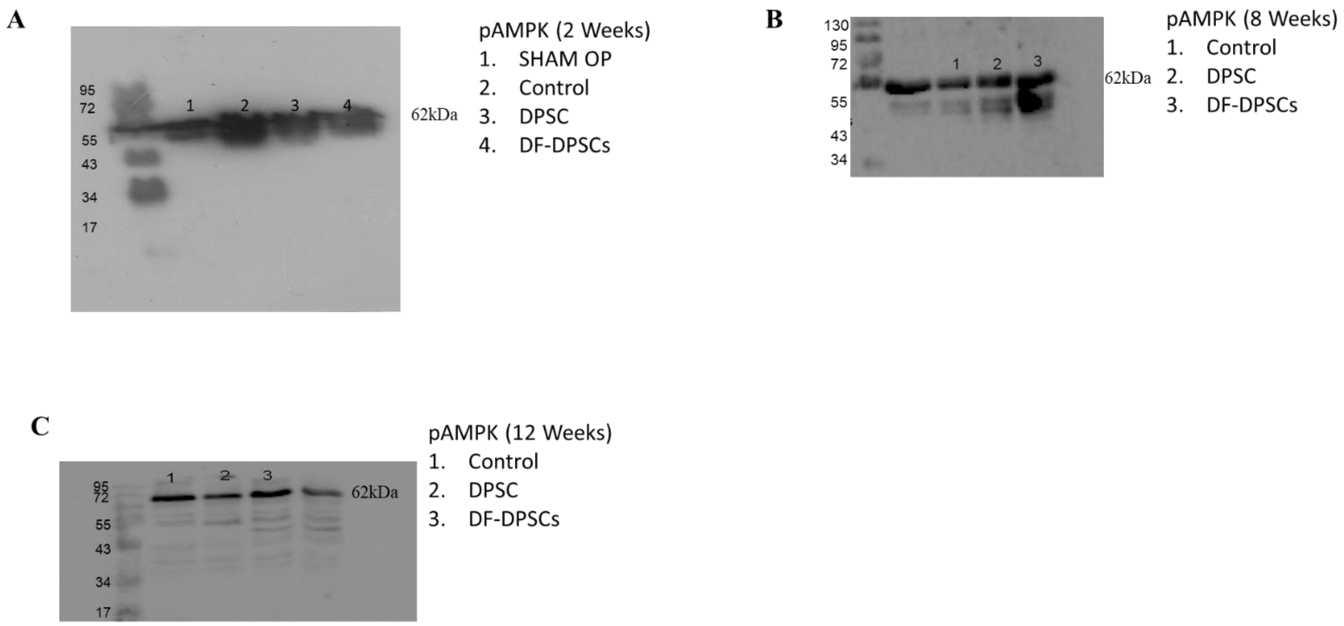

Figure 9. The whole gels immunoblot of pAMPK (62kDa) protein expressions at (A) 2 (B) 8 and (C) 12 weeks after surgery in rat brain. The bands were cropped at the exact kDa and are shown in the main manuscript and quantified using ImageJ software, and the differences are represented by a histogram.

Figure 10

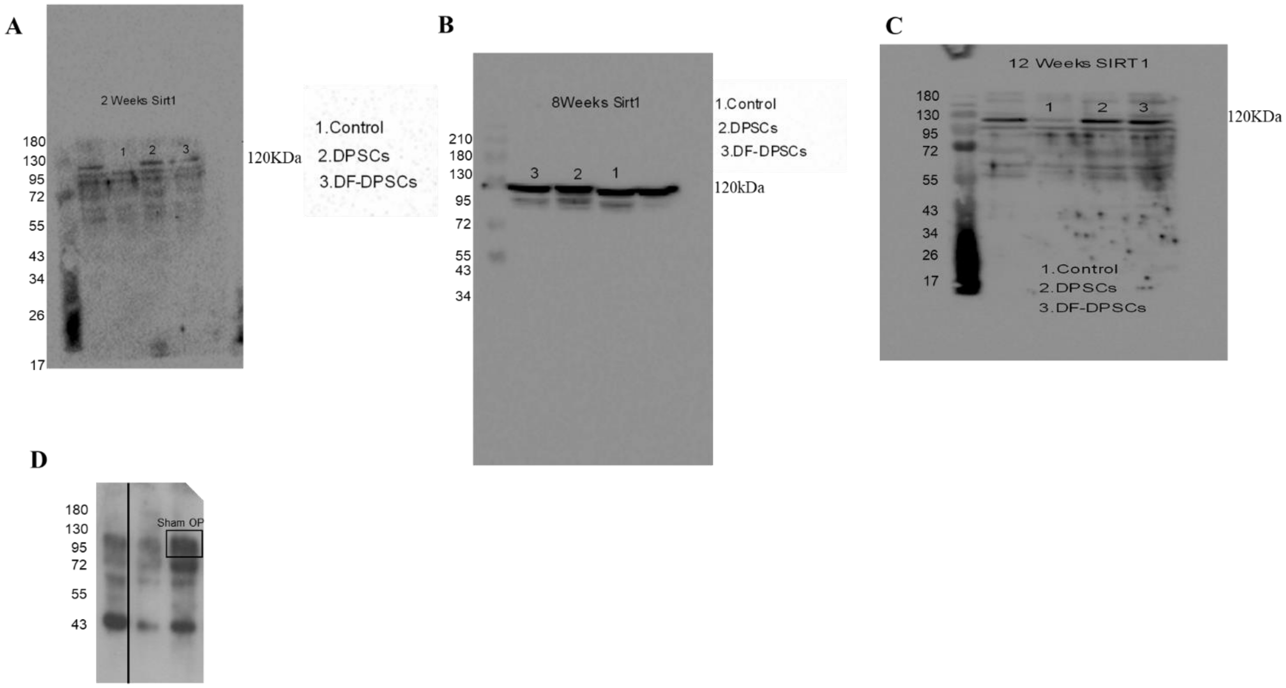

Figure 10. The whole gels immunoblot of Sirt1 (120kDa) protein expressions at (A) 2 (B) 8 and (C) 12 weeks after surgery in rat brain. The bands were cropped at the exact kDa and are shown in the main manuscript and quantified using ImageJ software, and the differences are represented by a histogram. (D) represents expression of Sirt1 in SHAM OP.

Figure 11

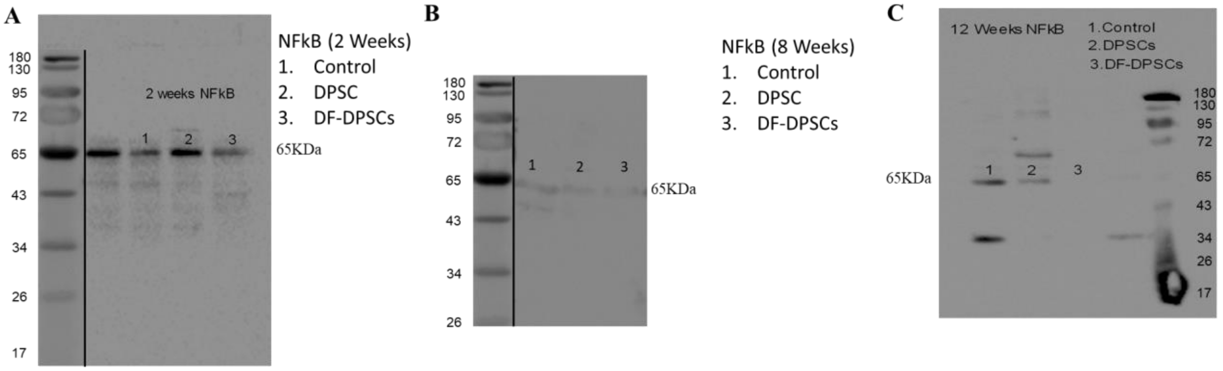

Figure 11. The whole gels immunoblot of NFkB (65kDa) protein expressions at (A) 2 (B) 8 and (C) 12 weeks after surgery in rat brain. The bands were cropped at the exact kDa and are shown in the main manuscript and quantified using ImageJ software, and the differences are represented by a histogram.

Note: Here we used two methods for final development i.e. X-ray development and the Bio-Rad Gel Doc EZ machine.

The following proteins were developed on X-ray film manually

β-Actin, Iba1 (12weeks), GFAP (8, 12 weeks), IL-1 *β*, TNF-α, IL-4, TGF-*β*, pAMPK (2 weeks), NFkB (8 weeks)

The following proteins were developed using Bio-Rad Gel Doc EZ

Iba1 (2, 8 weeks), GFAP (2 weeks), 8-OXO, pAMPK (8, 12 weeks), Sirt1, NFkB (2, 12 weeks)

To provide an ease for readers, we adjusted all blot background to gray color while the exposure and contrast were kept same for all blots.
